# Erythema exsudativum multiforme imitierendes IgA-positives Anti-p200-Pemphigoid

**DOI:** 10.1007/s00105-026-05642-0

**Published:** 2026-01-21

**Authors:** Greta Ancker, Chiara L. Blomen, Nina Booken, Ute Siemann-Harms, Stefan W. Schneider

**Affiliations:** Klinik für Dermatologie und Venerologie, Universitätsklinikum Hamburg-Eppendorf, Martinistr. 52, 20246 Hamburg, Deutschland

**Keywords:** Bullöse Dermatosen, Blasenbildende Autoimmundermatose, Laminin-γ1, Laminin-β4, Immunfluoreszenz (direkt/indirekt), Bullous dermatosis, Blistering autoimmune dermatosis, Laminin-γ1, Laminin-β4, Immunofluorescence (direct/indirect)

## Abstract

Das Anti-p200-Pemphigoid ist eine seltene subepidermale bullöse Autoimmundermatose, die sich klinisch variabel präsentieren und damit andere blasenbildende Dermatosen imitieren kann. Wir berichten über einen 65-jährigen Patienten mit ausgeprägten palmoplantaren Bullae und Vesikulae sowie Schleimhautbeteiligung, welche in der Zusammenschau an ein Erythema exsudativum multiforme (EEM) erinnerten. Die weiterführende Diagnostik ergab in der direkten Immunfluoreszenz (DIF) lineare IgA-, IgG- und C3-Ablagerungen entlang der dermoepidermalen Junktionszone. Mittels indirekter Immunfluoreszenz (IIF) auf NaCl-separierter humaner Spalthaut („salt-split skin“) wurden IgA- und IgG-Antikörper im Bereich des Blasenbodens nachgewiesen. Im Immunoblot konnten Autoantikörper gegen das p200-Antigen sowie Laminin-β4 nachgewiesen werden. Erst dadurch konnte die Diagnose eines IgA-positiven Anti-p200-Pemphigoids gestellt werden, einer sehr seltenen und bislang kaum beschriebenen Variante des Anti-p200-Pemphigoids. Das präsentierte Fallbeispiel unterstreicht somit die Bedeutung des Algorithmus aus Histologie, DIF und IIF zur korrekten Diagnosestellung bei bullösen Dermatosen.

## Abstract (en)

Anti-p200 pemphigoid is a rare subepidermal blistering dermatosis with variable clinical manifestations that can mimic other bullous skin diseases. We report the case of a 65-year-old patient presenting with extensive bullae on the palms and soles, as well as mucosal erosions. These findings were initially consistent with a diagnosis of erythema multiforme. Direct immunofluorescence (DIF) revealed linear IgA, IgG, and C3 antibody deposits along the dermoepidermal junction. While indirect immunofluorescence (IIF) on salt split skin demonstrated IgA and IgG antibodies binding on the dermal side of the split, immunoblot analysis revealed the presence of antibodies against the 200-kDa protein p200 as well as against laminin-β4. Based on these findings, the diagnosis of IgA-positive anti-p200 pemphigoid, a very rare variant barely described in literature, was made. This case highlights the importance of thorough differential diagnosis in blistering diseases and emphasizes the diagnostic value of immunohistochemical techniques.

## Anamnese

Wir berichten über einen 65-jährigen Patienten, der sich aufgrund von neu aufgetretenen bullösen Hautveränderungen sowie oralen und genitalen Schleimhauterosionen, begleitet von ausgeprägtem Juckreiz und Schmerzen, in unserer Klinik vorstellte. Außer einer arteriellen Hypertonie bestanden keine weiteren Vorerkrankungen. Der Patient erhielt 2 Wochen zuvor Impfungen gegen Influenza, SARS-CoV‑2 und Varizella Zoster.

## Befund

**Abb. 1:**
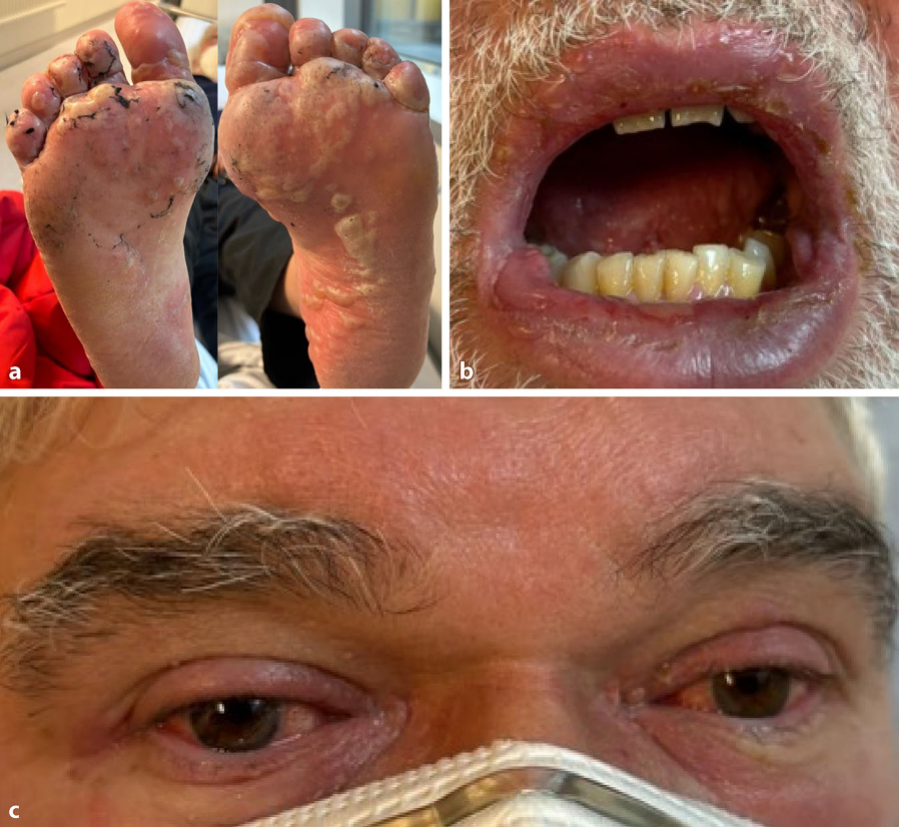
Klinische Präsentation bei Vorstellung. **a** Bullae der Fußsohlen, **b** enorale Erosionen, **c** konjunktivale Injektion und periorbitales Ödem

Klinisch imponierten plantar pralle, serös gefüllte Bullae (Abb. 1a) sowie palmar derb tastbare, erythematöse Papulovesikel und Plaques. Axillär zeigten sich multiple, teilweise anulär stehende, serös gefüllte Vesiculae auf erythematösem Grund neben sekundär erodierten Hautläsionen. Begleitet wurden die Hauteffloreszenzen durch enorale Erosionen (Abb. 1b) und beidseitige konjunktivale Injektion (Abb. 1c). In der Zusammenschau des klinischen Erscheinungsbildes (kokardenförmige Anordnung, Schleimhautbeteiligung) und der Anamnese (zuletzt erfolgte Impfungen) wurde initial die Verdachtsdiagnose eines Erythema exsudativum multiforme (EEM) gestellt.

## Diagnostik

Ein Abstrich aus dem Blaseninhalt ergab keinen Nachweis pathogener Erreger. In der histologischen Aufarbeitung einer Hautbiopsie zeigte sich eine subepidermale Spaltbildung mit neutrophilen Granulozyten und vereinzelten eosinophilen Infiltraten. Die direkte Immunfluoreszenz (DIF) ergab lineare IgG-, IgA- und C3-Ablagerungen entlang der dermoepidermalen Junktionszone. In dem Screeningtest der indirekten Immunfluoreszenz (IIF) auf NaCl-separierter humaner Spalthaut („salt-split skin“) wurden IgG- und IgA-Antikörper am Boden der artifiziellen Blase nachgewiesen. Mittels Immunoblot konnten Antikörper gegen das 200-kDa-Protein p200 sowie gegen Laminin-β4 detektiert werden, sodass abschließend die Diagnose eines IgA-positiven Anti-p200-Pemphigoids gestellt wurde.

## Therapie und Verlauf

Initial wurde neben einer topischen Therapie eine Steroidstoßtherapie (Prednisolon 1 mg/kgKG) begonnen. Trotz ergänzter immunmodulierender Therapie mit Dapson kam es im Rahmen des stationären Aufenthaltes zu weiterem Hinzutreten von Vesiculae, sodass eine notfallmäßige Therapie mit intravenösen Immunglobulinen (IVIG) eingeleitet wurde. Hierunter zeigte sich eine rasche Regredienz der bullösen Dermatose und der Schleimhautbeteiligung. Langfristig konnte unter Erhaltungstherapie mit Dapson eine Restitutio ad integrum erreicht werden.

## Diskussion

Das Anti-p200-Pemphigoid ist eine seltene subepidermale bullöse Autoimmundermatose, die erstmalig 1996 beschrieben wurde [1] und sich klinisch variabel präsentieren kann. Differenzialdiagnostisch abzugrenzen sind insbesondere das bullöse Pemphigoid, das Erythema exsudativum multiforme im Falle einer kokardenförmigen Anordnung der Effloreszenzen sowie einer Schleimhautbeteiligung, im Falle von IgA-Positivität die lineare IgA-Dermatose und darüber hinaus aufgrund der Antikörperablagerung am Blasenboden die Epidermolysis bullosa acquisita (EBA) [2].

Das Anti-p200-Pemphigoid ist durch intensiven Juckreiz gekennzeichnet. Es kann mit einer Beteiligung der Schleimhäute sowie typischerweise der Hände und Füße einhergehen, die Läsionen können kokardenförmig angeordnet sein. Auf diese Weise kann es das EEM imitieren [3]. Zeigt sich im Screeningtest der IIF die Antikörperbindung am Blasenboden der artifiziellen Blase, so kann der Verdacht auf ein Anti-p200-Pemphigoid erhärtet werden. Die korrekte Diagnosestellung gelingt durch den Antikörpernachweis gegen die entsprechenden Zielantigene p200, Laminin-γ1 [4] und/oder, wie kürzlich identifiziert, Laminin-β4 [2, 5, 6]. Der Nachweis von IgA-Antikörpern ist bei dem Anti-p200-Pemphigoid sehr selten und bislang kaum beschrieben [7].

Die exakte Diagnosestellung einer bullösen Dermatose gelingt häufig nur unter kombinierter Betrachtung des klinischen Bildes und gezielter immunhistologischer Diagnostik einschließlich der direkten und indirekten Immunfluoreszenz.

## Fazit für die Praxis

- Das Anti-p200-Pemphigoid kann andere blasenbildende Dermatosen imitieren.
- Insbesondere bei einer mit ausgeprägtem Juckreiz einhergehenden palmoplantaren Manifestation und Schleimhautbeteiligung sollte differenzialdiagnostisch an ein Anti-p200-Pemphigoid gedacht werden.
- Unklare blasenbildende Dermatosen erfordern ein mehrstufiges diagnostisches Vorgehen, bestehend aus Histologie, DIF und IIF einschließlich des Screening- („salt-split skin“) und Bestätigungstests (Immunoblot).
